# A Ranked Sparsity Extension to the Bayesian Information Criterion: A Tool for Selecting Variables from Multiple Data Modalities

**DOI:** 10.3390/e28090943

**Published:** 2026-08-22

**Authors:** Ryan A. Peterson, Sarah M. Bird, Logan M. Harris, Patrick J. Breheny, Joseph E. Cavanaugh

**Affiliations:** 1 Department of Internal Medicine, Carver College of Medicine, University of Iowa, 375 Newton Road, Iowa City, IA 52242, USA; 2 Department of Biostatistics, College of Public Health, University of Iowa, 145 N. Riverside Dr., Iowa City, IA 52242, USA; 3 Department of Biostatistics and Informatics, University of Colorado–Anschutz Medical Campus, 13001 E. 17 Ave., Aurora, CO 80031, USA

**Keywords:** derived variables, feature selection, information criteria, interactions, model selection, multimodal data

## Abstract

The concept of ranked sparsity, originally introduced in the context of penalized regression, arises in modeling applications when an expected disparity exists in the quality of information between different feature sets. Its presence can cause traditional and modern model selection methods to fail because such procedures commonly presume “covariate equipoise”—that each potential parameter is equally worthy of entering into the final model. However, this presumption does not always hold, especially in the presence of derived variables or with highly disparate feature sets (i.e., multi-modal data). For instance, when all possible interactions are considered as candidate predictors, the sheer number of them grossly inflates the number of false discoveries, resulting in unnecessarily complex and difficult-to-interpret models with many (truly spurious) interactions. In this work, we motivate a ranked sparsity extension to the Bayesian Information Criterion (RBIC) that requires a stronger level of evidence in order to allow certain variables (e.g., interactions vs main effects and genetic vs clinical covariates) into a model. We compare the performance of RBIC relative to competing methods for selecting polynomials and interactions in a simulation study and in two applications, showing that stepwise selection guided by RBIC produces better-predicting, more transparent models (with fewer false interactions) compared to existing alternatives.

## 1. Introduction

### 1.1. Background—Sparsity and Skepticism

When selecting a model, statisticians must traditionally balance many trade-offs. We are constantly trying to balance simplicity with predictive accuracy, parsimony with complexity, sparsity with saturation, and bias with variance. While we have a multitude of tools at our disposal, in most realistic situations, the reliability of these instruments can change given information we cannot access. Consider sparsity, for instance. A model can have many parameters, but a *sparse* model can only have a handful by definition. Does nature produce mostly sparse models? How could we possibly know this? Often times, we can understand more about a mechanism, or predict more accurately, by collecting new features. Perhaps, if we collect enough features about a generating mechanism, the remaining stochasticity might completely vanish, allowing us to predict newly generated data *perfectly*.

Putting the merits of determinism aside, we often take for granted that scientists know when to *stop* considering new features. With the advent of new computational methods and theory that help sift through arbitrarily large sets of features, scientists are understandably becoming increasingly ambitious in this regard. However, this Icarean ambition to include as many features as possible, while well-intentioned, tends to drive down the proportion of variables that contain real signal, ultimately leaving the scientific endeavor dead in the water. (In Greek mythology, Icarus escaped from a prison on the island of Crete by means of wings that his father, a master craftsman, constructed from feathers and wax. Despite his father’s warning, Icarus flew too close to the sun, and the heat caused his wings to melt. He fell and drowned in the Icarean Sea.)

We define the “sparsity level” as the proportion of candidate variables (a.k.a., features, covariates, or predictors) that are inactive in the true generating model. Conversely, the “saturation level” is defined as the proportion of candidate variables that are active. The sparsity level is governed by a mix of what cannot be known about nature’s true generating model and what can (sometimes) be known about the ambition of a particular scientific project.

It is certainly easier to *describe* sparse models; with a postulated sparse (or *parsimonious*) model, we can be more precise, collect less data, and improve the interpretability of our final model. And we do have tools to help us decide between sparse or saturated models: model selection criteria. Here, we define such criteria broadly as measures that are used to evaluate whether a certain predictor should be included in the model or not. These criteria typically have a penalty term, which acts as a “cost-of-admission” for each predictor (i.e., predictors are required to add a certain level of improvement in fit in order to be admitted into the model). These popular tools come with many trade-offs we will describe. Bootstrapping and cross-validation (CV) are increasingly being used to overcome the limitations of traditional model selection criteria (most importantly the asymptotic assumptions). However, these methods can be cumbersome, both computationally and in terms of their interpretation.

In practice, the goal of an analysis often dictates which method/criterion is used to select a final model. If a sparse model is desired, the Bayesian Information Criterion (BIC) [1] can be used. If prediction is the only concern, the Akaike Information Criterion (AIC) [2] or CV can be used. Once it is recognized that we cannot realistically know whether the true model is sparse or not, this real trade-off (that depends on the true generating mechanism) is relegated to the background of practicality, where a glossed-over presumption is made about the generating mechanism: a prior belief that all of the covariates are equally worthy of entering into the model. We refer to this presumption as “covariate equipoise.” In situations where multiple levels of sparsity can be expected among covariates, we will show how the presumption of covariate equipoise can have detrimental effects on every one of these model selection tools. With this impetus, we have developed several simple solutions to this problem for many settings—settings which we refer to as “ranked sparsity”. A related technique for this problem, the Sparsity-Ranked Lasso (SRL), is a useful regularization-based approach for selecting interactions and polynomials. In this work, we present a separate, information-criterion-based ranked-sparsity methodology: the Ranked-sparsity extension to BIC (RBIC).

### 1.2. A Motivating Example

The intuition behind ranked sparsity is that in many cases, covariates should not be presumed to be created equal. Consider the following simplistic example. A research team wishes to predict a response *y* using 20 covariates and 500 observations. Carefully investigating each covariate’s functional form and examining residual diagnostics, their statistician selects several of the 20 covariates as important predictors in a model that optimally fits the data interpretably. However, wondering if better predictions might be possible, the team also tries some state-of-the-art technology to train an army of machine learning (ML) models that they use for an ostensibly unbeatable (though completely opaque) predictive model. Sure enough, on new data, the predictions from the ensemble are more accurate than those from the statistical model. Let us consider why this can happen.

One key factor is that the army of ML models is not exactly concerned about treating each covariate equally. In trying to predict *y*, each member of this ML army (each *weak learner*) is free to grab from an urn of practically infinite predictors (e.g., random splits of each covariate interacted with other random splits of each covariate). The ensemble (the *super learner*) then observes which ones work best and uses weights, tuning parameters, and extra-sample validation to cleverly aggregate the results and optimize them for prediction. With 20 predictors in her original bin of candidates (not including additional polynomial and spline terms), the statistician did not consider any interactions between covariates. After all, in order to check all pairwise interactions, she would have had to sift through 190 more predictors, each of which would have made the model’s interpretation more nuanced. To consider three-way interactions renders the selection process increasingly Icarean; there are 1140 of those! If she were to consider all possible interactions of any order, she would have to consider ∑i=12020i=1,048,575 candidate variables!

Given this explosion of derived interactions, it is no wonder that the prevailing guidance is to consider an interaction in a model-selection framework only when there is substantive evidence to justify it beforehand. Harrell, for instance, notes that higher-order interactions are typically ignored unless specified *a priori* based on knowledge of the subject matter and recommends pre-specifying the small set of plausible interactions rather than testing all of them [3]. In other words, absent such justification, we assume *a priori* that nature’s generating mechanism is completely sparse in terms of interactions. However, when we zoom out and look at science more broadly, how do scientists ever identify a clinically relevant interaction if the convention is never to model one without prior justification?

This heuristic is a tacit acknowledgment of ranked sparsity: *most* of the signal in a linear regression problem can be found without considering interactions, and so the full set of possible interactions tends to add little to no signal (and a great deal of noise). This presumption nonetheless sacrifices a non-negligible amount of signal that flexible machine learning methods can often recover, albeit at the cost of interpretability. Statisticians are hamstrung because model selection tools do not work well when there is a large differential in the sparsity level among candidate predictors; the performance of model selection criteria is directly tied to the “true” sparsity level. In Appendix A, we illustrate how model selection criteria with higher penalties work better in situations with a high sparsity level, whereas ones with low penalties work better in situations with a high saturation level, demonstrating that the optimal penalty depends greatly on the true sparsity level in the covariate space. Therefore, when there is an *expected* disparity in sparsity among candidate predictors (such as interactions vs. main effects), model selection tools can and should account for this by using different penalties.

Our goals in this paper are as follows. After describing our notation and providing necessary background on existing tools, we motivate ranked sparsity methodology for covariate groups of different sizes and interactions, first by tying the performance of penalization in model selection criteria to the true saturation level, then by arguing why the expected saturation level for interactions and large feature sets should generally be presumed to be lower. Next, we identify RBIC as a novel extension of BIC that can be used to select from multiple covariate groups (and interactions) with different expected sparsity levels. We compare its performance to other methods in a simulation study and in two real data applications and describe its user-friendly implementation via the ICtoolkit R package(version 0.1.2). We conclude with a discussion.

### 1.3. Traditional Model Selection Criteria

For a data generating model G:x→y, where x is a vector of *p* covariates, the goal of model selection is to build a linear model to predict future *y* observations via $f\left(\beta ,x\right)=x^{T}\beta$. Many model selection criteria have been explored over the course of the last century as solutions to this problem, and most can be broadly classified as either *consistent* or *efficient* [4]. Consistent model selection criteria, including, for example, BIC [1], the minimum description length criterion (MDL) [5], and Extended BIC [6], aim to select the most parsimonious model with a requisite structure; as n→∞, a consistent criterion is guaranteed to select the correct model (with probability one), under the strong assumption that this model is included in the candidate collection [7]. In contrast, efficient model selection criteria are *not*, in general, consistent. Rather than recover a true structure, they aim to minimize the error of the model when predicting the response for new data; an efficient criterion will select a model whose predictive risk converges to that of the best candidate model available, in the sense that the ratio of the two tends to 1 as n→∞ [8]. Crucially, this benchmark is the best *available* model, so unlike consistency, efficiency does not presume that the true model lies within the candidate set [9]. Efficient model selection criteria include, for example, AIC [2], small-sample-corrected AIC (AICc) [10], and Mallows’ Cp [11].

When should we use an efficient criterion, and when should we use a consistent one? When should we abandon them both? Besides the goals of a particular project, other major factors that come into play for this decision include (but are not limited to) the following: the sample size *n* (how much data we collected), the number of candidate predictors *p* (how ambitious we are), and the number of true predictors *s* (the number of truly active candidate predictors). With this notation, the sparsity level is $1-\frac{s}{p}$, and the saturation level is $\frac{s}{p}$. As p→n, the asymptotic assumptions on which many model selection criteria are based become increasingly violated and their performance tends to suffer as a result. As $\frac{s}{p}↓$ (i.e., the saturation level decreases), the performance will also vary by penalization (efficient vs. consistent criteria) in ways we explore in Appendix A, but first we introduce model search frameworks.

### 1.4. Searching for the Correct Model with Information Criteria

A model selection criterion can only be calculated for a model that has been fit, which is not a problem for small *p* where all of the possible combinations of the *p* predictors can be quickly fit and optimized over (a process called “best-subsets” selection). However, in some cases (when *p* is large and/or when *n* is massive), it becomes impractical to fit all candidate models, the number of which increases on the order of $2^{p}$. In lieu of fitting every possible model, one can use a stepwise approach. In forward stepwise (greedy) selection, the starting point is a null model. In the first step (k=1), univariate models are fit for each feature and selected from by using an information criterion (IC). Then, at each subsequent step *k*, models with the remaining p−k+1 features are fit, their IC values computed, and the best one selected. The process stops when all of the newly fit models increase the IC. Backwards stepwise selection is similar, but its starting point is the full model, and covariates are taken out one-at-a-time until the IC stops improving. Finally, a forwards and backwards approach consists of checking not only for the addition of new features but also the removal of features that had been included in a previous step.

Stepwise approaches are considerably faster than best-subsets, but they still take a lot of computation time in high-dimensional or massive *n* settings (this will become evident in our second application). In these settings, the Least Absolute Shrinkage and Selection Operator (the lasso) [12] becomes especially useful. The lasso simultaneously estimates coefficients for each of the *p* features and selects from them, such that they are either “active” (i.e., ${\hat{\beta }}_{j}\neq 0$), or inactive ${\hat{\beta }}_{j}=0$. The estimated non-zero coefficients do suffer from a bias that is introduced by the lasso’s penalty term λ, but this bias is often warranted as it significantly cuts down on the variance of having too saturated of a model.

With features $\left[x_{1},x_{2},\ldots ,x_{p}\right]=X_{n\times p}$, and a centered response variable y, where some (but not all) features are related to y, the lasso solution can be obtained by minimizing the following expression with respect to β:

$${\left\|y-X\beta \right\|}^{2}+\lambda \sum_{j=1}^{p}\left|\beta_{j}\right|.$$

The lasso is not a final solution to choosing an optimal model; a model must be selected along the *coefficient path*: a sequence of λ values that fall upon a one-dimensional grid. In other words, the tuning parameter that dictates how much to shrink the non-zero coefficients, λ, must be selected somehow. This is where AIC, BIC, and CV come back into play; before this point in the process, the lasso search has been completely informed by goodness-of-fit (subject to shrinkage). In this context, all of the same properties related to model saturation hold for these criteria as was discussed in the previous section.

It is important to distinguish the difference between model search methods (best-subsets, stepwise approaches, and the lasso) and model selection criteria. The former is used to search the model space, while the latter is used to choose the optimal model from a set of candidates. Both are necessary, although currently, the only available tools for ranked sparsity focus on the search process. Earlier work [13] introduced the Sparsity Ranked Lasso, but this only addresses the search aspect of the model selection process. Here, we take up the question of incorporating ranked parsimony into the selection process as well.

### 1.5. The Optimal Penalty Depends on the Saturation Level

To ground this intuition empirically, we conducted a simulation (presented in full in Appendix A) comparing AIC, BIC, and cross-validation (CV) for tuning lasso models across a range of true saturation levels ω=s/p, at a fixed sample size n=250 and with increasing numbers of candidate predictors *p*. The central finding is that no single fixed penalty performs well across saturation regimes: BIC-tuned models predict best when the truth is sparse, AIC-tuned models prevail as saturation grows, and the gap between them widens as p→n, where AIC’s performance deteriorates markedly; cross-validation performs well throughout by selecting the penalty from extra-sample performance. A companion simulation holding the signal-to-noise ratio fixed showed the same deterioration of AIC as p→n, with the criterion-to-criterion contrast attenuated once individual coefficients approached the noise floor (i.e., small enough that the signal is difficult to distinguish from sampling variability).

The implication is that when distinct groups of candidate predictors are expected *a priori* to have different saturation levels, a model selection criterion should apply different levels of penalization to those groups rather than a constant level. This is the central idea of ranked sparsity, which we develop next.

## 2. Ranked Sparsity—Motivation

In the previous section, we illustrated how AIC and BIC perform very differently for different sparsity levels when the sample size is held constant, and that this difference increases with the number of candidate predictors. The performance of a penalty is highly dependent on the sparsity level. Now, we turn our attention to cases where we anticipate there being a different sparsity level across all of the covariates.

### 2.1. For Covariate Groups of Different Sizes

Say we have *K* covariate sets of varying sizes $p_{k}$. These covariate groups might include a small group of clinical features, a large group of genomic features, and another even larger group of methylation markers, as was the case in VanHawkins et al. [14]. When covariates come from different groups or modalities, we typically expect different levels of sparsity for each group. A substantial literature already exploits known group structure in the regularization framework: the group lasso [15] penalizes coefficients group-wise so that a group enters or leaves the model as a unit, and the sparse-group lasso [16] combines this with a within-group penalty to produce sparsity within as well as between groups. These methods, however, use group structure to decide *which groups act together* and were not developed to encode a prior belief that covariates in some groups should face a higher bar for entry than covariates in others. In such cases, one could utilize a technique like the IPF-lasso [17] to perform cross-validation separately across all groups. However, this process is computationally expensive and requires K−1 additional tuning parameters to properly penalize each group according to its saturation level. An alternative approach is to use ranked sparsity methods such as the sparsity-ranked lasso [13]. The SRL uses a Bayesian motivation to naturally penalize groups of covariates according to their dimension size; in VanHawkins et al. [14], the SRL performed as well as IPF-lasso without the need for additional group-specific tuning.

Specifically, Bayesian interpretation of the lasso solution involves characterizing each $\beta_{j}$ as having independent and identical prior double exponential (Laplace) distributions, such that their joint prior density is

$$\pi \left(\beta \right)=\prod_{j=1}^{p}\frac{\lambda }{2}e^{-\lambda |\beta_{j}|}.$$

Peterson and Cavanaugh [13] showed that when the covariates are part of distinct groups of disparate sizes $p_{k}$, we can modify this prior to have group-specific $\lambda_{k}$ values, such that the joint prior density becomes

$$\pi \left(\beta \right)=\prod_{k=1}^{K}\prod_{j=1}^{p_{k}}\frac{\lambda_{k}}{2}e^{-\lambda_{k}\left|\beta_{j}\right|}.$$

When $\lambda_{k}=\lambda \sqrt{p_{k}}$ (which can be readily set up using penalty weights), larger groups are naturally penalized more heavily than smaller groups so that the prior information contributed to the posterior by each group is held constant across groups. This property is desirable when we expect larger groups to be more sparse, as it ties the penalization directly to the group dimension. However, this method is only applicable in penalized regression settings; additional tools are required for this purpose in other model selection settings (e.g., best-subsets or stepwise selection). We will return to this in Section 3.2.

### 2.2. For Interactions

Take now the context of selecting from all possible pairwise interactions. We define “main effects” as linear coefficients in a model and “interaction effects” as coefficients on the product of covariates that correspond to the main effects. In this section, we will show that the maximum saturation level attainable for the interaction effects is necessarily limited by hierarchy assumptions about the true generating model. Model hierarchy is typically broken down into “strong”, e.g., $y_{i}=\beta_{0}+\beta_{1}x_{1i}+\beta_{2}x_{2i}+\beta_{3}x_{1i}x_{2i}$, “weak”, e.g., $y_{i}=\beta_{0}+\beta_{1}x_{1i}+\beta_{3}x_{1i}x_{2i}$, and “anti” hierarchical models, e.g., $y_{i}=\beta_{0}+\beta_{3}x_{1i}x_{2i}$.

Let $s_{M}$ again refer to the number of signals in the main effects of $p_{M}$ possible main effect predictors, and let $s_{I}$ refer to the number of signals in the interactions of pI=pM2 possible interactions. Also, let $\omega_{M}$ and $\omega_{I}$ refer to the saturation levels in the main effects and the interactions, respectively. Assume that $p_{M}\geq 2$ and that $p_{M}\;\;\omega_{M}\in Z$. By definition, we have that $s_{M}=p_{M}\;\;\omega_{M}$ and sI=pM2ωI, and therefore

(1) ωI=sIpM2.

Under strong hierarchy, the signals in the interactions must be combinations of the signals in the main effects, which implies

(2) sI≤pMωM2.

Together, (1) and (2) imply

maxωI=pMωM2pM2=ωM(pMωM−1)pM−1.

**Figure 1:**
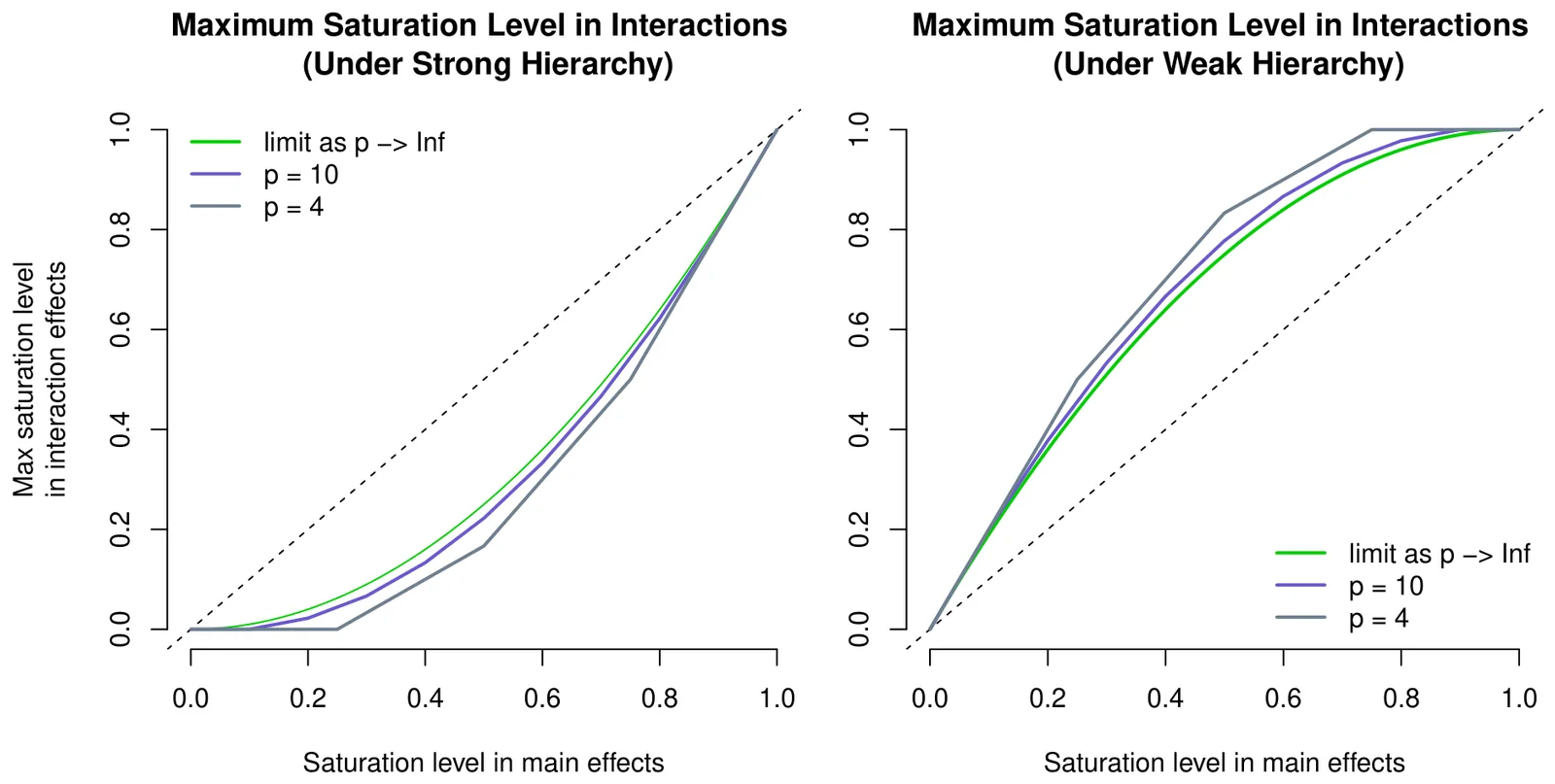
Theoretical upper bound on the saturation level of pairwise interaction effects, $\max\omega_{I}$, as a function of the saturation level of the main effects, $\omega_{M}$, under strong hierarchy (**left**) and weak hierarchy (**right**). Each panel shows the bound for p=4 and p=10 candidate main effects, together with its limiting form as p→∞; the dashed line marks equality, $\omega_{I}=\omega_{M}$. Under strong hierarchy the interactions are always sparser than the main effects ($\max\omega_{I}<\omega_{M}$); under weak hierarchy the bound still depends on the main-effect signals but may rise above the dashed line. In both regimes the attainable interaction saturation is constrained by the main effects, motivating a heavier penalty on interaction terms than on main effects.

This upper bound is plotted as a function of $\omega_{M}$ in Figure 1 (left), and it is evident from the figure that $\max\omega_{I}<\omega_{M}\;\forall \;\omega_{M}\in \left(0,1\right)$. (Using L’Hôpital’s rule, we know that $\lim_{p_{M}\to \infty }\max\omega_{I}=\omega_{M}^{2}$.) Under weak hierarchy, instead of the expression in (2), we have

$$s_{I}\leq \sum_{j=1}^{s_{M}}p_{M}-j=\frac{1}{2}p_{M}\omega_{M}\left(2p_{M}-p_{M}\omega_{M}-1\right).$$

The corresponding bound on the saturation level of the interactions then becomes

maxωI=12pMωM(2pM−pMωM−1)pM2 =ωM(2pM−pMωM−1)pM−1.

We see that in the weakly hierarchical case, the maximum saturation level in the interactions is still bounded by the signals in the main effects, though it is not restricted to be less than the saturation level in the main effects (Figure 1, right). Under the heredity principle of Chipman [18], strongly hierarchical structures are the most probable *a priori*, weakly hierarchical ones less so, and anti-hierarchical ones the least. The bounds above trace this same ordering: under strong hierarchy the interaction saturation is provably below the main-effect saturation; under weak hierarchy it remains tied to the main-effect signals, though no longer strictly below them; and under anti-hierarchy the main-effect constraint disappears altogether. These derivations therefore suggest that one should not apply the same penalty to both main effects and interactions and expect optimal performance: because ranked sparsity concerns the expected, *a priori* interaction saturation, and the prior mass arguably concentrates on the more hierarchical regimes, we should still expect interactions to be sparser than main effects even though any particular generating model need not obey this.

Crucially, a heavier penalty like those we propose for ranked sparsity raises the evidentiary bar for interactions and higher-order terms but does not forbid them. A weakly or anti-hierarchical truth can therefore still be recovered when warranted by the data. This distinction separates ranked sparsity from the established literature on structured interaction selection, which largely enforces hierarchy as a hard constraint. Strong-heredity penalized regression [19], the hierarchical group-lasso of glinternet [20], and the regularization path algorithm RAMP [21] all restrict the feasible set so that an interaction may enter only when its parent main effects are present, while hierNet [22] imposes hierarchy through a convex constraint on the coefficient matrix. These approaches are well justified where hierarchy genuinely holds, but because they assign zero prior mass to anti-hierarchical truths, no amount of evidence can recover one. Ranked sparsity instead expresses hierarchy as a *preference* such that interactions are admitted, but at a steeper price, which keeps the anti-hierarchical corner of the model space reachable while still protecting against the multiplicity that makes it dangerous.

### 2.3. Conclusion

We have shown that the optimal penalty for any model selection criterion is highly dependent on the true sparsity level. We have also shown that when there are different groups of covariates with different expected sparsity levels, as is especially true when main effects are considered alongside interactions, it is important to account for this in the model selection process. In the next section, we explore how to do this with a novel ranked sparsity method, RBIC.

## 3. Ranked Sparsity via RBIC

### 3.1. The Extended Bayesian Information Criterion

The Extended Bayesian Information Criterion (EBIC) of Chen and Chen [6] extends BIC to cases in which the candidate pool *p* is large relative to the sample size. One of the standard conditions for BIC’s consistency is that *p* is fixed; EBIC adds a term that grows with *p* to restore consistency when *p* increases with *n*, and in particular when $p>\sqrt{n}$, where BIC is otherwise inconsistent. By placing a uniform prior on all candidate models, BIC induces a strongly nonuniform prior on model *size*, since there are pm candidate models of size *m*; this combinatorial factor concentrates sharply near m=p/2 as *p* grows. BIC therefore encodes an implicit prior that the saturation level ω=m/p is near 0.5, which is rarely a reasonable prior belief. We refer the reader to Peterson and Cavanaugh [23] for visualizations of this prior and additional background. EBIC corrects this prior imbalance by penalizing models in proportion to the size of their model class. Letting *m* denote the dimension of a candidate model with parameter vector β,

EBIC=−2logl(β^)+mlogn+2γlogpm =BIC+2γlogpm.

**Figure 2:**
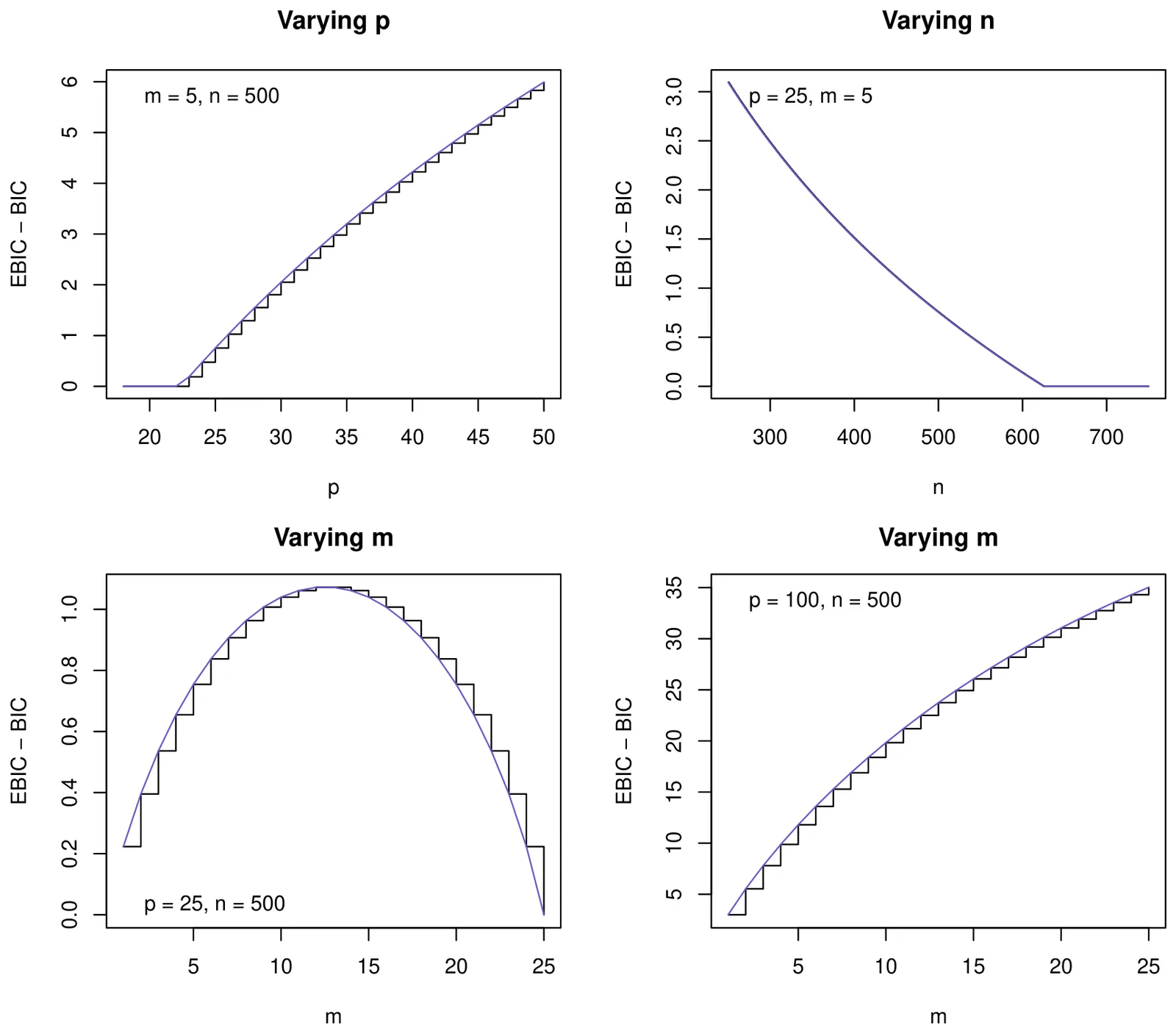
The additional penalty that EBIC imposes over BIC, 2γEBIClogpm, shown as a function of the candidate-pool size *p*, the sample size *n*, and the model size *m*. Panels vary, in turn, *p* (**top left**), *n* (**top right**), and *m* ((**bottom row**), at p=25 and p=100); the remaining quantities are held fixed at the values given in each legend. Larger values on the vertical axis indicate a heavier penalty relative to BIC. The extra penalty grows with *p*, shrinks with *n* (reaching zero once $p\leq \sqrt{n}$, where BIC is already consistent), and is largest near m=p/2. Because *p*, *m*, and *n* are integer-valued, the black step functions give the exact penalty while the blue lines linearly interpolate between adjacent values.

The tuning parameter γ controls the strength of EBIC’s correction, interpolating between BIC’s implicit prior and a uniform prior on model size; Peterson and Cavanaugh [23] visualize how γ reshapes that prior. Three cases are useful. If γ=0, EBIC reduces to the original BIC. If γ=1, EBIC places a uniform prior on the marginal distribution of model size and is consistent whenever *p* grows polynomially with *n* ($p=O(n^{\kappa })$ for any κ≥0 not depending on *n*). Intermediate values trade between these extremes; for example, γ=0.5 retains consistency for κ<1. Setting γ=1 can be too stringent in practice, so Chen and Chen [6] propose a data-adaptive choice that targets consistency without being unnecessarily aggressive: solving γ=1−1/(2κ) from $p=n^{\kappa }$ and clamping at zero,

$$\gamma_{\mathrm{EBIC}}=\max\;\left(\frac{\log(p/\sqrt{n})}{\log p},\;0\right),$$

which equals zero when $p\leq \sqrt{n}$ (the regime in which BIC is already consistent) and rises smoothly with *p*. The corresponding additional penalty over BIC is 2γEBIClogpm. Figure 2 shows how this term varies with *p*, *n*, and *m*: it grows with *p*, decays with *n* (eventually to zero), and is largest near m=p/2, which means that for high-*p* scenarios the penalty is effectively increasing with *m* for sparse models.

An intuitive framing of EBIC is to consider each candidate covariate as a ball in a single urn of size *p*, where any candidate model is a random draw of *m* balls, giving pm possible models of that size. In the next section, we extend this one step further to the setting of *K* groups of covariates.

### 3.2. Ranked Sparsity Extension for BIC

#### 3.2.1. Description and Motivation

We use this same “balls and urns” metaphor to intuitively motivate a ranked sparsity extension of BIC (RBIC). Instead of one urn with *p* covariates, we postulate that we have multiple (*K*) urns, each with $p_{k}$ covariates. Then, for any candidate model with $m_{k}$ parameters of $p_{k}$ possible for all k∈{1,2,…,K},

RBIC=−2logl(β^)+∑k=1Kmklogn+∑k=1K2γklogpkmk =BIC+∑k=1K2γklogpkmk,0≤γk≤1∀k.

This formulation of RBIC effectively penalizes a covariate’s coefficient differently depending on which covariate subspace (or urn) it comes from. In the case of pairwise interactions, the penalty (when p>2) is higher for interaction effects than for main effects.

By default, we suggest setting $\gamma_{k}=\gamma_{\mathrm{EBIC}}$ to benefit from its consistency property for high-dimensional data:

RBIC=BIC+2γEBIC∑k=1Klogpkmk.

Setting $\gamma_{k}=0\;\forall \;k$ yields the original BIC, and if K=1, RBIC is identical to EBIC. In Application 1, we will sweep $\gamma_{k}$ from 0 to $\gamma_{\mathrm{EBIC}}$ to illustrate how its performance changes.

An alternative is to apply the rule of Chen and Chen [6] within each urn separately, yielding *per-group* values

$$\gamma_{k}=\frac{\log(p_{k}/\sqrt{n})}{\log p_{k}}\;1\left(p_{k}\geq \sqrt{n}\right).$$

Whereas the default ties every group’s penalty to the dimension of the full candidate pool, the per-group specification localizes this factor to the urn from which each covariate is drawn: small urns (those with $p_{k}<\sqrt{n}$, where BIC is already consistent) revert to BIC’s penalty, while large urns retain an essentially EBIC-strength correction. Because larger urns thereby receive systematically larger $\gamma_{k}$, this specification widens the ranked-sparsity differential between groups. We denote this variant RBIC-pg and evaluate it alongside the default specification in the studies that follow. When K=1, RBIC reduces exactly to EBIC and therefore inherits its selection-consistency guarantees [6]. For K>1, we conjecture that analogous guarantees hold when each $\gamma_{k}$ satisfies the Chen-Chen rate condition with respect to its own group dimension. However, establishing this property in general is complicated by the fact that the *K* candidate pools may grow at different asymptotic rates $p_{k}=O\left(n^{\kappa_{k}}\right)$, making the conditions for selection consistency depend on the relative differences among the $\kappa_{k}$ as well as how the true signal is distributed across groups.

#### 3.2.2. Implementation

For any likelihood-based model with well-specified covariate groups, RBIC is amenable to model selection via best-subsets or any stepwise selection framework. However, several advancements in the computational efficiency of information-criterion-based model searching, such as those implemented by the R packages leaps [24] and bestglm [25], are unavailable. These packages take advantage of the fact that all models with *m* parameters have the exact same penalty (mlogn for BIC, or 2m for AIC). With RBIC, each covariate may be penalized differently, so not all models with *m* parameters have the same penalty. We have developed the R package ICtoolkit [26], publicly available at https://github.com/petersonR/ICtoolkit (accessed on 10 August 2026), to provide a unified interface for computing AIC, AICc, BIC, EBIC, and RBIC. The package supports models of class lm, glm, glmnet [27], and ncvreg [28]. For lm and glm objects, each criterion returns a scalar value. For penalized regression objects (glmnet and ncvreg), the criteria are computed across the full regularization path, returning a vector of values (one per λ), enabling information-criterion-based tuning parameter selection as an alternative to cross-validation.

The RBIC implementation in ICtoolkit accepts a user-specified grouping structure via a named list P_index, where each element is a character vector of variable names belonging to that group. For example, if main effects belong to one group and pairwise interactions to another, the user specifies this as P_index = list(main = c("x1", "x2", …), interactions = c("x1:x2", …)); this can be automated with the expand_scope function. The package then computes the group-specific combinatorial penalties logpkmk for each group *k* and applies the appropriate γ value. The default γ is the data-adaptive rule of Chen and Chen [6], though the package also supports per-group γ values, fixed numeric values, and user-supplied functions.

For stepwise model selection, ICtoolkit provides an ic_step() function that supports all five criteria as well as forward, backward, and bidirectional search for lm and glm objects. The implementation mostly extends the approach of MASS::stepAIC [29], except that stepAIC enforces the marginality principle when proposing moves (an interaction becomes a candidate only once its main effects are in the model), whereas ic_step() evaluates every single-term addition permitted by the scope at each step, implementing a hierarchy *preference* expressed through the RBIC penalty rather than a hierarchy *constraint*. Consequently, the two functions produce identical selections for AIC or BIC when the scope contains no hierarchically related terms; when interactions or polynomials are in scope, they can differ. Admitting non-hierarchical models can also change the stepwise behavior in a manner that is dependent on where covariates are centered; therefore, unless a predictor’s raw origin is scientifically meaningful, we recommend centering and scaling predictors before expansion. When the criterion is set to EBIC or RBIC, ic_step() applies the appropriate extended penalties at each candidate evaluation step.

As described in Section 1.4, we can also use information criteria to select the overall tuning parameter λ in a lasso-based selection framework as long as covariate groups are specified (in this case we will use BIC, EBIC, and RBIC). For penalized models, ICtoolkit computes the criterion at every point along the regularization path, and the optimal λ is selected as the value that minimizes the chosen criterion.

#### 3.2.3. Simulation Study

In order to study how RBIC compares to its competitors, we simulate data under the Friedman generating model [30], shown below.

$$y=10\sin\left(\pi x_{1}x_{2}\right)+20{\left(x_{3}-0.5\right)}^{2}+10x_{4}+5x_{5}+N\left(0,3^{2}\right)$$

In addition to these 5 “signal” terms, 5 noise variables were generated as well (all independent uniform random variables). All ten covariates were standardized (centered and scaled) before the polynomial and interaction candidate terms were constructed, per the recommendation in the previous subsection. For 10,000 Monte Carlo simulations, models were fit to n=80 observations using forwards-and-backwards stepwise regression using BIC, EBIC, RBIC, and RBIC-pg.

Under the quadratic scope the candidate pool contains P=65 terms, and RBIC uses K=3 groups (10 main effects, 10 squared terms, and 102=45 pairwise interactions). Under the cubic scope, P=195 and K=5 groups, with two new groups added (10 cubed terms and 103=120 three-way interactions). Thus, $\gamma_{\mathrm{EBIC}}\approx 0.048$ for the linear scope, $\gamma_{\mathrm{EBIC}}\approx 0.475$ for the quadratic scope, and $\gamma_{\mathrm{EBIC}}\approx 0.584$ for the cubic scope, whereas RBIC-pg applies $\gamma_{k}\in \left\{0.048,0.424,0.542\right\}$ for groups of size 10, 45, and 120, respectively. Note that in the cubic scope P=195 substantially exceeds n=80, so this setting provides an empirical glimpse at a high-dimensional scenario.

We investigate how RBIC performs in the linear regression setting (no candidate polynomials or interactions), the quadratic regression setting (squared terms and pairwise interactions), and the cubic regression setting (cubed terms and three-way interactions). Note that in the linear regression setting, K=1, so RBIC and EBIC are identical. To quantify model performance, we calculate the root-mean-squared prediction error (RMSPE), defined as $\sqrt{N^{-1}\sum_{i=1}^{N}{\left(y_{i}-{\hat{y}}_{i}\right)}^{2}}$ over N=10,000 newly generated observations, as well as the number of false negatives (NFN), the number of false positives (NFP), and the coefficient-wise selection percentage. Based on the form of the Friedman model, the covariates with legitimate non-zero coefficients are $x_{1}$, $x_{2}$, $x_{4}$, $x_{5}$, $x_{1}^{2}$, $x_{2}^{2}$, $x_{3}^{2}$, and the pairwise interaction $x_{1}*x_{2}$; all other covariates, if selected, were considered false positives.

Table 1 reports the means and standard deviations of these performance metrics across simulations, and Table 2 shows the per-variable selection percentages. As expected, BIC works well only in the linear regression setting but fails badly in the quadratic or cubic regression setting (in terms of prediction and the number of false positives). BIC’s poor performance is due to the violation in asymptotic assumptions as the number of candidate predictors is higher than the sample size for these settings. EBIC and RBIC both control the number of false positives and enjoy similarly better prediction performance, though RBIC saw a small but consistent gain in predictive efficacy relative to EBIC for both quadratic and cubic regression. This improvement is accompanied by a slightly lower number of false negatives but with little change in the number of false positives between EBIC and RBIC. All methods suffered from higher RMSPE when considering spurious cubic terms, with RBIC suffering comparably less than EBIC and BIC.

The per-group specification RBIC-pg, which computes each group’s γ from its own dimension, adds a further refinement. Because the polynomial groups here are equal in size to the main effects (ten candidates each), RBIC-pg applies the same small correction to them while retaining a strong penalty on the much larger interaction pool. The consequence is visibly higher recovery of the true squared terms: in the quadratic setting RBIC-pg selects $x_{1}^{2}$, $x_{2}^{2}$, and $x_{3}^{2}$ in 62%, 62%, and 92% of simulations, versus 53%, 52%, and 86% for RBIC and 44%, 43%, and 81% for EBIC (Table 2), with a correspondingly lower number of false negatives. This improved recovery of legitimate polynomial terms comes at the cost of an increase in false positives among the derived terms (for example, a cubic-scope NFP of 3.3 versus RBIC’s 2.1), leaving RBIC-pg essentially tied for the best predictive performance of any criterion in the quadratic setting (3.84 versus RBIC’s 3.85) and a close second to RBIC in the cubic setting. The false-positive rate remains tightly controlled for all extended BIC-family criteria across all scopes (roughly 1–2% in the cubic scope, versus BIC’s 22%).

In summary, RBIC’s ranked sparsity penalty provides an improvement in predictive performance relative to EBIC when applied to interactions, especially as the candidate pool becomes more complex and higher-dimensional (i.e., with more interactions).

#### 3.2.4. Simulation Study II: Increasing Interaction Density

The Friedman-model study fixes the number of active interactions at one. To investigate the robustness of RBIC under richer interaction structures, we replicate the interaction simulation design used to evaluate the sparsity-ranked lasso [13]. This simulation set-up will assess the sensitivity of RBIC’s preference for main effects when the interaction set becomes dense enough that it is no longer sparse relative to the main effects in absolute terms. For each of S=1000 replicates, we generate n=300 observations on p=20 independent uniform (0,1) covariates, of which s=5 carry active main effects with normally distributed coefficients scaled so that they collectively sum to 10 in absolute value. Among the 202=190 candidate pairwise interactions, b∈{0,1,…,10} are active, with coefficients summing to $10\sqrt{12/7}$ (the scaling accounting for the variance of a product of uniform covariates). This scaling ensures that the per-group overall signal-to-noise ratio remains approximately constant and that the signal is spread across the active main effect and interaction terms. Active interactions are drawn under a mixed hierarchy following Chipman’s paradigm [18]: an interaction whose “parent” main effects are both active (i.e., a strongly hierarchical interaction) is most probable (probability 0.7), one active parent (i.e., a weakly hierarchical interaction) less so (0.2), and no active parents least so (0.1). When b>5, the number of active interactions exceeds the number of active main effects (5), inverting the ranked-sparsity ordering in absolute counts.

Models were selected by forwards-and-backwards stepwise regression from the null model using AIC, BIC, EBIC, RBIC, and RBIC-pg as implemented in ICtoolkit, with the candidate scope comprising all main effects and pairwise interactions (210 terms). For RBIC we consider the default γ specification ($\gamma_{\mathrm{EBIC}}\approx 0.47$), fixed values γ∈{0.25,1}, and the *per-group* specification (here, γ≈0.05 for the 20 main effects and γ≈0.46 for the 190 interactions). Unless otherwise indicated, results ascribed to RBIC are under the uniform $\gamma =\gamma_{\mathrm{EBIC}}$ specification. Performance is measured by the RMSPE on 10,000 newly generated observations, and by the numbers of false positives and false negatives, computed separately for main effects and interactions.

**Figure 3:**
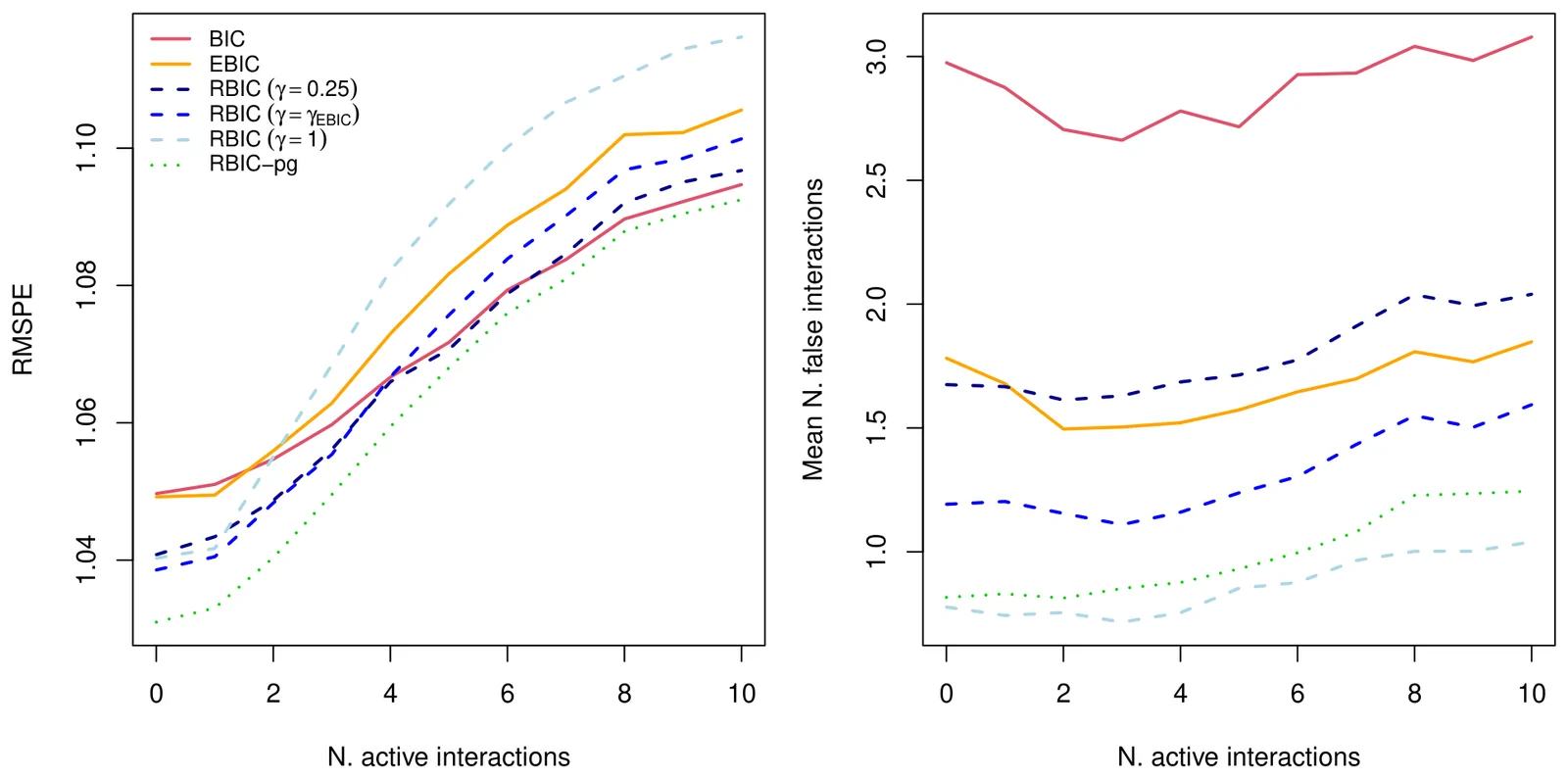
Performance across different true interaction-densities (*b* active interactions, S=1000 replicates per setting). (**Left**): mean RMSPE. (**Right**): mean number of falsely selected interaction terms. AIC is omitted from both panels for scale; its RMSPE runs roughly 15% higher, and it selects roughly 26–30 false interactions per fit (see Table 3).

Figure 3 presents predictive performance, and Table 3 reports selection metrics across different numbers of active interactions *b*. Three findings stand out. First, AIC dramatically over-selects interactions at every density level (26–30 false interactions per fit, with RMSPE about 15% higher than the other ICs), echoing the behavior of the all-pairwise lasso documented in Peterson and Cavanaugh [13]. Second, RBIC variants all perform well at low *b*, but the more conservative ones with higher γ predict increasingly worse relative to BIC when *b* increases, with the exception of RBIC-pg, which interestingly predicts superlatively at each candidate *b*. RBIC-pg also attains the second fewest falsely selected interactions of any method at every density level, losing only to the most conservative RBIC (γ=1). The large number of false interactions selected by BIC are emblematic of the ranked sparsity issue: BIC does not penalize interactions any more or less than main effects. In summary, this study supports some level of robustness of RBIC to different interaction structures and suggests the per-group γ specification as a sensible default for interaction selection. In the next section, we apply RBIC to real data sets to illustrate how it can be used in practice.

## 4. Application

### 4.1. Application 1: Interaction Selection in COVID-Era ICU Caregiver Data

#### 4.1.1. Description

We illustrate RBIC’s utility for interaction selection using data from Amass et al. [31], who surveyed family members of patients admitted to the ICU during the COVID-19 pandemic (n=298). The outcome is the Impact of Event Scale (IES) total score, a continuous measure of post-traumatic stress. We consider 10 candidate main-effect predictors: four continuous variables (age, SOFA score, Charlson comorbidity index, and ICU length of stay) and six categorical variables (sex, ethnicity, race, education, patient death, and psychotropic medication use). Race and education are multi-level factors contributing 3 degrees of freedom each; all other categorical predictors are binary.

To test whether RBIC appropriately controls the inclusion of spurious complex terms, we compare forward stepwise selection under two candidate scopes:

- **Two-way scope**: all main effects, quadratic polynomials of the continuous variables, and all 102=45 pairwise interactions (59 terms, 103 df total).
- **Three-way scope**: the 2-way scope augmented by all 103=120 3-way interactions (179 terms, 399 df total).

The key question is whether expanding the candidate pool from 2-way to 3-way interactions causes complex terms to proliferate without improving model performance.

#### 4.1.2. Group Structure for RBIC

Candidate terms are grouped by their structural complexity, with group sizes reflecting degrees of freedom rather than term counts (since multi-level factor terms consume more than one degree of freedom each). Under the 2-way scope, K=2 groups are defined: main effects ($p_{1}=14$ df) and 2-way scope terms comprising pairwise interactions and quadratic polynomials ($p_{2}=89$ df). Under the 3-way scope, a third group is added for three-way interactions ($p_{3}=296$ df). Since the term pools are substantially larger for the 2-way and 3-way scopes, RBIC’s combinatorial penalty logpkmk is correspondingly steeper for interactions, encoding the ranked sparsity prior that complex terms should face a higher bar for inclusion.

Because γ controls the strength of RBIC’s penalty relative to BIC, with γ=0 recovering BIC, we report across $\gamma \in \{0,0.10,0.25,\gamma_{\mathrm{EBIC}}\}$, with the same γ applied uniformly across groups. Note that the default $\gamma_{\mathrm{EBIC}}$ is computed from the total candidate dimension of the scope in use, so it differs between scopes: ≈0.385 under the 2-way scope (P=103 df) and ≈0.524 under the 3-way scope (P=399 df). The γ=0 case (denoted RBIC.00) serves as a reminder that RBIC reduces to BIC; the intermediate values RBIC.10 and RBIC.25 interpolate between BIC and the default RBIC, allowing us to characterize how RBIC’s performance changes as the analyst dials the strength of the ranked-sparsity penalty. We additionally report the per-group specification RBIC-pg, for which the rule yields γ≈(0,0.37) under the 2-way scope and γ≈(0,0.37,0.50) under the 3-way scope. Because the main-effect pool ($p_{1}=14$ df) falls below $\sqrt{n}\approx 17.3$, RBIC-pg penalizes main effects exactly like BIC while applying progressively stronger corrections to the larger complex-term groups.

#### 4.1.3. Results

Table 4 presents the average model characteristics and held-out predictive accuracy across 100 random 2/3-1/3 train/test splits, for each criterion under both candidate scopes. First, we notice that AIC overfits substantially in both scopes, most catastrophically under the 3-way scope where it selects a three-way interaction on every split and incurs a mean test RMSPE of 2190.5 (compared to roughly 5.8 for the BIC-family criteria). Second, among the BIC-family criteria, as γ increases from 0 (BIC) through 0.10, 0.25, and to each scope’s default $\gamma_{\mathrm{EBIC}}$, the mean degrees of freedom, the percentage of splits selecting a three-way interaction, and the model’s permissiveness toward complex terms all decrease monotonically. Among the uniform-γ variants, RBIC at γ=0.10 achieves the lowest mean test RMSPE (5.848 in the 2-way scope; 5.855 in the 3-way scope) while selecting fewer parameters than BIC and roughly halving the three-way selection rate (7% vs. 13%). Under the 2-way scope, RBIC.25, default RBIC, and EBIC all incur higher mean test RMSPE than BIC, suggesting they are under-selecting active signal effects. Under the 3-way scope, those same heavier penalties suppress three-way selection almost entirely (3%, 1%, and 0%, respectively, vs. BIC’s 13%).

The per-group specification performs best of all: RBIC-pg attains the lowest mean test RMSPE in the table (5.808 under both scopes), making RBIC-pg the only criterion completely insensitive to this candidate-scope expansion. All other considered penalties involve the total candidate dimension, meaning that their penalties shift with the scope even when no three-way term is selected, and their performance suffers when drastically increasing the candidate scope. As a side benefit, the per-group rule removes the need to tune γ at all in this application: by construction it applies no extra penalty to the main effects (whose pool falls below $\sqrt{n}$) and near-EBIC-level penalties to the interaction groups, which here delivered the best of both worlds—BIC-like permissiveness toward main effects alongside EBIC-like discipline on complex terms.

### 4.2. Application 2: Multimodal Survival Modeling in Lung Adenocarcinoma

#### 4.2.1. Description

Whereas Application 1 examined ranked sparsity along the *complexity axis* (main effects vs. interactions), this application demonstrates RBIC on the *modality axis*: two covariate groups of vastly different dimension that arise from distinct data sources. We use the lung adenocarcinoma data of Shedden et al. [32], a multi-site study of n=442 patients with gene expression profiling and clinical annotation. The outcome is overall survival (time to death or censoring; 236 events). The candidate predictors comprise two groups:

- **Clinical** ($p_{1}=7$ df): age (centered and scaled), sex, adjuvant chemotherapy status, smoking history (never vs. ever), tumor grade (3 levels, 2 df), and race (White vs. non-White). Missing values are imputed via *k*-nearest neighbors; categorical predictors are dummy-coded.
- **Genetic** ($p_{2}=22,283$): Affymetrix HG-U133A microarray probe-set expression levels, centered and scaled to unit variance.

The total candidate pool is $P=p_{1}+p_{2}=22,290$. The extreme asymmetry ($p_{2}/p_{1}\approx 3183$) makes this a prototypical multimodal setting in which standard model selection criteria, which assume covariate equipoise, will be overwhelmed by the genetic group. In the main text we focus on forward stepwise selection under BIC, EBIC, and RBIC (including the per-group specification RBIC-pg), which selects directly from the full candidate pool; a complementary regularization-path analysis (lasso and the Sparsity-Ranked Lasso) is deferred to Appendix B.

#### 4.2.2. Group Structure for RBIC

For RBIC, we define K=2 groups whose sizes match the number of candidate predictors in each modality: $p_{1}=7$ (clinical) and $p_{2}=22,283$ (genetic). Because $p_{2}\gg p_{1}$, the combinatorial penalty logpkmk is substantially steeper for genetic predictors than for clinical predictors at every model size, encoding the ranked sparsity prior that clinical covariates (which are fewer and typically well-motivated) should face a lower bar for inclusion.

With n=442 and a total candidate pool of P=22,290, $\gamma_{\mathrm{EBIC}}=\log\left(P/\sqrt{n}\right)/\log P\approx 0.696$, which EBIC applies to the pooled candidate set and the default RBIC applies to both groups. The per-group specification instead yields $\gamma_{1}=0$ for the clinical pool (since $p_{1}=7<\sqrt{n}\approx 21.0$) and $\gamma_{2}\approx 0.696$ for the genetic pool. Because the genetic group comprises all but seven of the candidates, its per-group value is very close to the pooled default.

#### 4.2.3. Results: Forward Stepwise Selection

Table 5 and Table 6 summarize the size and composition of each criterion’s stepwise-selected model. Forward stepwise selection under BIC resulted in a notably oversized model. By step 143, BIC had selected 141 genetic predictors and 2 clinical predictors before failing at step 144, likely due to near-multicollinearity among the large set of included genetic variables. This outcome is not unexpected. BIC does not account for the size of the search space and proceeds in a greedy, sequential manner. As the model grows large relative to the sample size, the asymptotic penalty underlying BIC becomes increasingly unreliable. The computational cost reflects this as BIC required approximately 14 h on 63 cores to reach this point. In contrast, EBIC and RBIC each completed selection in approximately 6 min, terminating after just 4 and 5 steps, respectively.

EBIC selected four variables in total: one clinical (age) and three genetic. RBIC selected five variables: three clinical (age, sex, and adjuvant chemotherapy) and two genetic. The key difference between the two criteria lies in the group-specific combinatorial penalty applied by RBIC, which imposes a heavier penalty on genetic predictors than on clinical ones, reflecting the prior assumption that genetic signal is sparse. As a result, RBIC is more permissive toward clinically meaningful predictors. Its selected clinical variables are well-established prognostic factors in this setting. Notably, EBIC and RBIC share two overlapping genetic markers, suggesting consistency in identifying the strongest genetic signals despite differences in penalization structure.

Because the clinical pool ($p_{1}=7$) falls below $\sqrt{n}$, RBIC-pg assigns it $\gamma_{1}=0$ (plain BIC), while the genetic pool receives $\gamma_{2}\approx 0.70$. Here, this produced the same five-variable model as the default RBIC (three clinical; two genetic). The two specifications coincide because the clinical signals (age, sex, and adjuvant chemotherapy) are strong enough to enter under either penalty, while the genetic group is so large that its per-group γ nearly matches the total-pool default. The agreement is reassuring: on this modality axis the RBIC family’s selections are robust to the choice between a common and a per-group γ.

A complementary analysis fits penalized Cox models along the regularization path (standard lasso and the Sparsity-Ranked Lasso) and selects the tuning parameter by each information criterion; those results, which are broadly consistent with the forward-stepwise selections reported here, are given in Appendix B.

The forward stepwise results using all criteria are presented in Table 6. BIC selected age and adjuvant chemotherapy, along with a large number of genetic predictors (138 genetic predictors in the table). EBIC identified age but not chemotherapy and also found three genetic markers (FAM117A, ZC2HC1A, and NDST1), reflecting EBIC’s conservative penalization. In addition to age and chemotherapy, RBIC also selected sex as a clinical variable alongside two genetic markers (FAM117A and ZC2HC1A). FAM117A appears in every model that includes any genetic variables regardless of criterion or method, suggesting that FAM117A carries consistent prognostic signal across selection approaches. This is further supported by the regularization results from Appendix B and previous analysis of the lung adenocarcinoma data, which found that FAM117A had a protective effect on survival [13].

## 5. Discussion

In this work, we have argued that “covariate equipoise,” the implicit prior that every candidate predictor is equally worthy of inclusion in a model, is built into AIC, BIC, and the lasso, and that this assumption breaks down whenever the analyst already has structural information that some covariates should face a higher bar than others. RBIC operationalizes this structure in the information-criterion framework: by partitioning the candidate pool into *K* groups and applying a group-specific combinatorial penalty logpkmk, RBIC requires increasingly stronger evidence for inclusion as a group’s pool grows. It is a natural cousin of the Sparsity-Ranked Lasso [13], which operates in the regularization framework, and of the IPF-lasso [17], which tunes group-specific $\lambda_{k}$ via cross-validation. Setting K=1 recovers Chen and Chen [6]’s EBIC; setting γ=0 recovers BIC. RBIC is therefore not a wholesale replacement for these criteria but a structured generalization that exposes assumptions previously hidden in their construction.

We have built an empirical case for that generalization across two simulation studies and two applications. The motivation is grounded in Appendix A: the optimal IC penalty depends sharply on the true saturation level, and no single criterion dominates. The Friedman simulation in Section 3.2.3 then showed that, when a polynomial/interaction expansion introduces candidates with a natural ranked-sparsity structure, RBIC consistently outperformed EBIC on prediction in both quadratic and cubic settings while preserving comparable false-positive control. The gain came specifically from the combinatorial term reflecting group structure. Application 1 traced this advantage on the *complexity* axis, where a γ sweep showed that RBIC at γ=0.10 retained most of BIC’s flexibility on main effects while roughly halving the spurious three-way interaction selection rate (7% versus BIC’s 13%) and predicting best among the uniform-γ variants. The per-group specification did better still, attaining the lowest mean test RMSPE of any criterion under both candidate scopes and, alone among the criteria considered, selecting the identical model whether or not the three-way pool was offered. Application 2 then demonstrated the *modality* axis, where the number of genetic covariates was over three orders of magnitude larger than the clinical pool. Forward stepwise under BIC blew up, admitting 141 genetic predictors before failing at step 144 after roughly 14 h of computation. RBIC and EBIC instead terminated quickly at parsimonious models: RBIC admitted three clinical predictors and two genetic ones; EBIC admitted only a single clinical predictor and three genetic. The per-group RBIC-pg selected the same five-variable model as the default RBIC, showing that the family’s selections here are robust to per-group versus uniform γ specification.

Our work has several limitations deserving attention. First, RBIC requires the group structure to be specified *a priori*; it operationalizes a prior conceptualization of a ranking but does not discover one. Further, the combinatorial penalty logpkmk is well defined when groups partition the candidate pool, but overlapping or nested groupings (a polynomial term that could plausibly belong either to a “main effect” or “complexity-1” group) require the analyst to commit to one assignment, and that decision can shape downstream results. In Application 2 for instance, RBIC is not directly able to incorporate different types of grouped genetic features such as pathways or gene networks because RBIC assumes the groups partition the candidate set; handling overlapping groups is a nontrivial extension. Second, the consistency results of Chen and Chen [6] extend to RBIC only via the K=1 reduction; a formal proof for K>1, particularly when group sizes grow at different rates with *n*, remains an open problem (see Section 3.2.1). Third, while we investigated two applications and two simulation studies, we did not find a single specification of γ that was empirically optimal across all settings; the best for a given application may need to be determined in context (e.g., based empirically on CV-based predictive accuracy or on the relative cost of missed vs false discoveries).

As a practical recommendation, the default $\gamma_{\mathrm{EBIC}}$ is a conservative-but-safe starting point that protects against false discoveries from the largest groups. In settings where prediction matters more than complete suppression of complex terms, exploring other uniform γ specifications may expose a useful middle ground. The per-group γ specification also seems to offer very good properties, as in Application 1 where it was completely robust to increasing the candidate scope from two-way to three-way interactions.

A further question, raised in review, concerns robustness. All of the criteria we consider are built on a maximized log-likelihood and are therefore only as robust as the likelihood supplied to them; in a setting with substantial unmodeled heterogeneity or heavy-tailed outcomes, a Gaussian working model may be unduly influenced by a handful of observations. However, this is a property of the goodness-of-fit term rather than of ranked sparsity itself. RBIC’s contribution lies entirely in the penalty: the group-specific term 2γ∑klogpkmk depends only on the candidate structure and the fitted model size, not on the form of the likelihood or on the residual distribution. Substituting a robust or heavy-tailed likelihood leaves the ranked-sparsity penalty untouched; Application 2, for instance, pairs the RBIC penalty with a Cox partial likelihood. However, in this work, we did not empirically evaluate combining RBIC with alternative likelihood specifications, so the finite-sample behavior of such combinations is a question we leave open.

These limitations suggest natural directions for future work. The most immediate is a data-driven choice of γ, either via cross-validation or an empirical-Bayes calibration; the per-group rule evaluated here fixes $\gamma_{k}$ from each group’s dimension alone, and a specification that adapts to an empirically validated saturation within each group may do better still. A formal consistency theory for K>1 would be valuable, especially in a framework that would incorporate regimes in which different groups grow at different polynomial rates. On the implementation side, the constant-penalty trick that makes leaps [24] and bestglm [25] efficient does not apply to RBIC, but a branch-and-bound algorithm with group-specific lower bounds appears tractable and would extend RBIC’s reach into best-subsets selection, building on efficient branch-and-bound best-subset implementations such as BranchGLM [33]. There are also clear conceptual neighbors worth exploring further. The modified BIC (mBIC) of Bogdan et al. [34] adds a multiplicity-aware term to BIC and arrives at a penalty closely related to EBIC’s combinatorial correction; RBIC’s group-specific motivation could be applied as a ranked extension of that same multiplicity adjustment to *K* pools of different size.

The broader contribution of this work is to make a hidden assumption visible. The implicit prior that every candidate covariate deserves the same admission penalty is rarely a justified scientific belief; more often it is an analytic convenience. When something is already known about how the candidate space is structured (interactions versus main effects; genetic versus clinical; derived versus measured), RBIC offers a modest tool for incorporating that knowledge explicitly. The cost is committing to a ranking; the benefit, as our applications and simulations have shown, is a shield against spurious complexity—one that lets the statistician fly as close to the sun as the data allow without burning their wings.

## Figures and Tables

**Table 1:** Performance of stepwise selection under BIC, EBIC, RBIC, and the per-group specification RBIC-pg for data simulated from the Friedman model (n=80). Each cell reports the mean (standard deviation) across simulations of the root-mean-squared prediction error (RMSPE), the number of false negatives (NFN), or the number of false positives (NFP). The linear, quadratic, and cubic labels denote the scope of candidate predictors: main effects only; main effects plus squared terms and pairwise interactions; and those plus cubic and three-way terms, respectively.

	BIC	EBIC	RBIC	RBIC-pg
**RMSPE**				
Linear	4.07 (0.18)	4.07 (0.18)	4.07 (0.18)	4.07 (0.18)
Quadratic	4.17 (0.44)	3.98 (0.37)	3.85 (0.33)	3.84 (0.33)
Cubic	6.94 (1.79)	4.38 (0.52)	4.09 (0.44)	4.16 (0.50)
**NFN**				
Linear	4.17 (0.41)	4.18 (0.42)	4.18 (0.42)	4.18 (0.42)
Quadratic	1.92 (0.94)	2.57 (1.16)	2.21 (1.04)	1.91 (0.89)
Cubic	2.35 (1.38)	4.12 (1.44)	2.98 (1.40)	2.71 (1.21)
**NFP**				
Linear	0.28 (0.52)	0.28 (0.52)	0.28 (0.52)	0.28 (0.52)
Quadratic	3.89 (2.58)	1.30 (1.43)	1.15 (1.31)	1.44 (1.48)
Cubic	41.27 (29.74)	2.17 (2.07)	2.08 (2.13)	3.32 (2.96)

**Table 2:** Per-variable selection percentages for the Friedman-model simulation (n=80), under BIC, EBIC, RBIC, and the per-group specification RBIC-pg. Each cell gives the percentage of simulations in which stepwise selection under the given criterion retained that variable, with rows grouped by candidate scope (linear, quadratic, or cubic). High percentages are desirable for the true signal variables listed; the final “Avg. FP rate” row instead reports the average false-positive rate, the mean selection percentage across the noise variables, for which lower is better.

	BIC	EBIC	RBIC	RBIC-pg
**Linear Regression**				
$x_{1}$	97.6	97.6	97.6	97.6
$x_{2}$	97.7	97.7	97.7	97.7
$x_{4}$	100.0	100.0	100.0	100.0
$x_{5}$	87.5	87.2	87.2	87.2
Avg. FP rate	4.7	4.6	4.6	4.6
**Quadratic Regression**				
$x_{1}$	98.1	94.9	97.8	98.4
$x_{2}$	98.1	95.1	97.9	98.5
$x_{4}$	100.0	99.9	100.0	100.0
$x_{5}$	90.8	80.8	89.7	92.1
$x_{1}^{2}$	59.8	44.1	52.6	62.4
$x_{2}^{2}$	60.0	42.9	51.8	62.0
$x_{3}^{2}$	91.0	81.0	86.3	92.2
$x_{1}\times x_{2}$	10.0	4.3	2.7	3.0
Avg. FP rate	6.8	2.3	2.0	2.5
**Cubic Regression**				
$x_{1}$	89.1	71.4	84.7	82.2
$x_{2}$	88.9	71.4	84.5	82.5
$x_{4}$	92.0	86.2	89.6	90.7
$x_{5}$	72.9	48.1	74.6	70.1
$x_{1}^{2}$	55.8	24.0	43.5	55.0
$x_{2}^{2}$	55.6	23.3	43.1	55.3
$x_{3}^{2}$	85.3	60.9	80.0	89.7
$x_{1}\times x_{2}$	25.5	2.3	2.2	3.5
Avg. FP rate	22.1	1.2	1.1	1.8

**Table 3:** Performance of stepwise selection across interaction-density settings (*b* active interactions; n=300, p=20, S=1000). Cells report the mean (standard deviation) across simulations. NFN and NFP denote the numbers of false negatives and false positives, split by main effects and interactions.

	AIC	BIC	EBIC	RBIC	RBIC-pg
b=0					
RMSPE	1.20 (0.11)	1.05 (0.03)	1.05 (0.03)	1.04 (0.03)	1.03 (0.02)
NFN (main)	2.1 (0.9)	2.2 (1.0)	2.6 (1.0)	2.1 (1.1)	1.6 (1.0)
NFN (int.)	0.0 (0.0)	0.0 (0.0)	0.0 (0.0)	0.0 (0.0)	0.0 (0.0)
NFP (main)	1.0 (1.2)	0.0 (0.2)	0.0 (0.1)	0.1 (0.2)	0.2 (0.4)
NFP (int.)	25.9 (14.2)	3.0 (1.8)	1.8 (1.3)	1.2 (1.2)	0.8 (1.0)
b=5					
RMSPE	1.23 (0.12)	1.07 (0.04)	1.08 (0.05)	1.08 (0.05)	1.07 (0.04)
NFN (main)	2.2 (0.9)	2.5 (1.0)	3.0 (1.0)	2.6 (1.1)	2.3 (1.0)
NFN (int.)	1.1 (0.8)	1.9 (0.9)	2.3 (1.0)	2.3 (1.0)	2.3 (0.9)
NFP (main)	1.2 (1.3)	0.1 (0.3)	0.1 (0.3)	0.1 (0.4)	0.3 (0.5)
NFP (int.)	28.5 (14.8)	2.7 (1.7)	1.6 (1.3)	1.2 (1.3)	0.9 (1.1)
b=10					
RMSPE	1.26 (0.12)	1.09 (0.04)	1.11 (0.04)	1.10 (0.04)	1.09 (0.04)
NFN (main)	2.3 (0.9)	2.8 (0.9)	3.2 (1.0)	2.9 (1.0)	2.5 (1.0)
NFN (int.)	4.3 (1.4)	6.6 (1.4)	7.2 (1.3)	7.4 (1.3)	7.5 (1.2)
NFP (main)	1.4 (1.4)	0.1 (0.3)	0.1 (0.3)	0.2 (0.4)	0.5 (0.7)
NFP (int.)	30.4 (14.5)	3.1 (1.8)	1.8 (1.4)	1.6 (1.4)	1.2 (1.2)

**Table 4:** Forward stepwise selection results for the COVID-era ICU caregiver data (n=298; outcome: Impact of Event Scale score), averaged over 100 random train/test splits (two-thirds train; one-third test). The two row blocks correspond to the 2-way candidate scope (main effects, quadratics, and pairwise interactions) and the 3-way scope (additionally including three-way interactions). RBIC.00, RBIC.10, and RBIC.25 are RBIC at fixed γ∈{0,0.10,0.25}, RBIC is the default $\gamma_{\mathrm{EBIC}}$ computed from each scope’s total candidate dimension (≈0.385 for the 2-way scope; ≈0.524 for the 3-way scope), and RBIC-pg is the per-group specification (γ=0 for main effects, ≈0.37 for 2-way terms, and ≈0.50 for 3-way terms). Mean df is the average number of parameters (excluding intercept) in the selected model; Test RMSPE is the root-mean-squared prediction error on the held-out third; % with 3-way is the percentage of splits selecting at least one three-way interaction.

Criterion	Mean df	2-Way	3-Way	Test RMSPE	SD	% with 3-Way
**2-way scope**						
AIC	23.7	6.4	-	6.176	0.459	-
BIC	4.7	0.9	-	5.853	0.323	-
RBIC.00	4.7	0.9	-	5.853	0.323	-
RBIC.10	4.4	0.8	-	5.848	0.309	-
RBIC.25	4.0	0.7	-	5.859	0.310	-
RBIC	3.5	0.5	-	5.875	0.305	-
RBIC-pg	3.6	0.2	-	5.808	0.317	-
EBIC	2.5	0.1	-	5.891	0.307	-
**3-way scope**						
AIC	47.1	2.8	7.8	2190.463	12,462.313	100%
BIC	4.8	0.9	0.1	5.871	0.332	13%
RBIC.00	4.8	0.9	0.1	5.871	0.332	13%
RBIC.10	4.4	0.7	0.1	5.855	0.310	7%
RBIC.25	4.0	0.7	0.0	5.860	0.309	3%
RBIC	3.2	0.4	0.0	5.888	0.307	1%
RBIC-pg	3.6	0.2	0.0	5.808	0.317	0%
EBIC	1.9	0.0	0.0	5.951	0.310	0%

**Table 5:** Forward stepwise selection results for Cox proportional hazards models on the Shedden lung adenocarcinoma data, under BIC, EBIC, RBIC, and the per-group specification RBIC-pg, drawing on all 22,290 candidate predictors (7 clinical; 22,283 genetic). Total is the number of predictors in the selected model, with Clinical and Genetic giving the breakdown by modality.

Criterion	Total	Clinical	Genetic
BIC	143	2	141
EBIC	4	1	3
RBIC	5	3	2
RBIC-pg	5	3	2

**Table 6:** Variables selected for the Shedden lung adenocarcinoma data by each combination of selection criterion (BIC, EBIC, RBIC, and RBIC-pg) via forward stepwise selection; ‘X’ marks a variable selected. Each displayed variable was selected by multiple criterion-method combinations; BIC additionally selected many other genetic predictors that are omitted here.

Variable	BIC	EBIC	RBIC	RBIC-pg
**Clinical**				
Age	X	X	X	X
Sex			X	X
Adj. Chemo	X		X	X
**Genetic**				
FAM117A	X	X	X	X
ZC2HC1A	X	X	X	X
NDST1	X	X		

**Figure A1:**
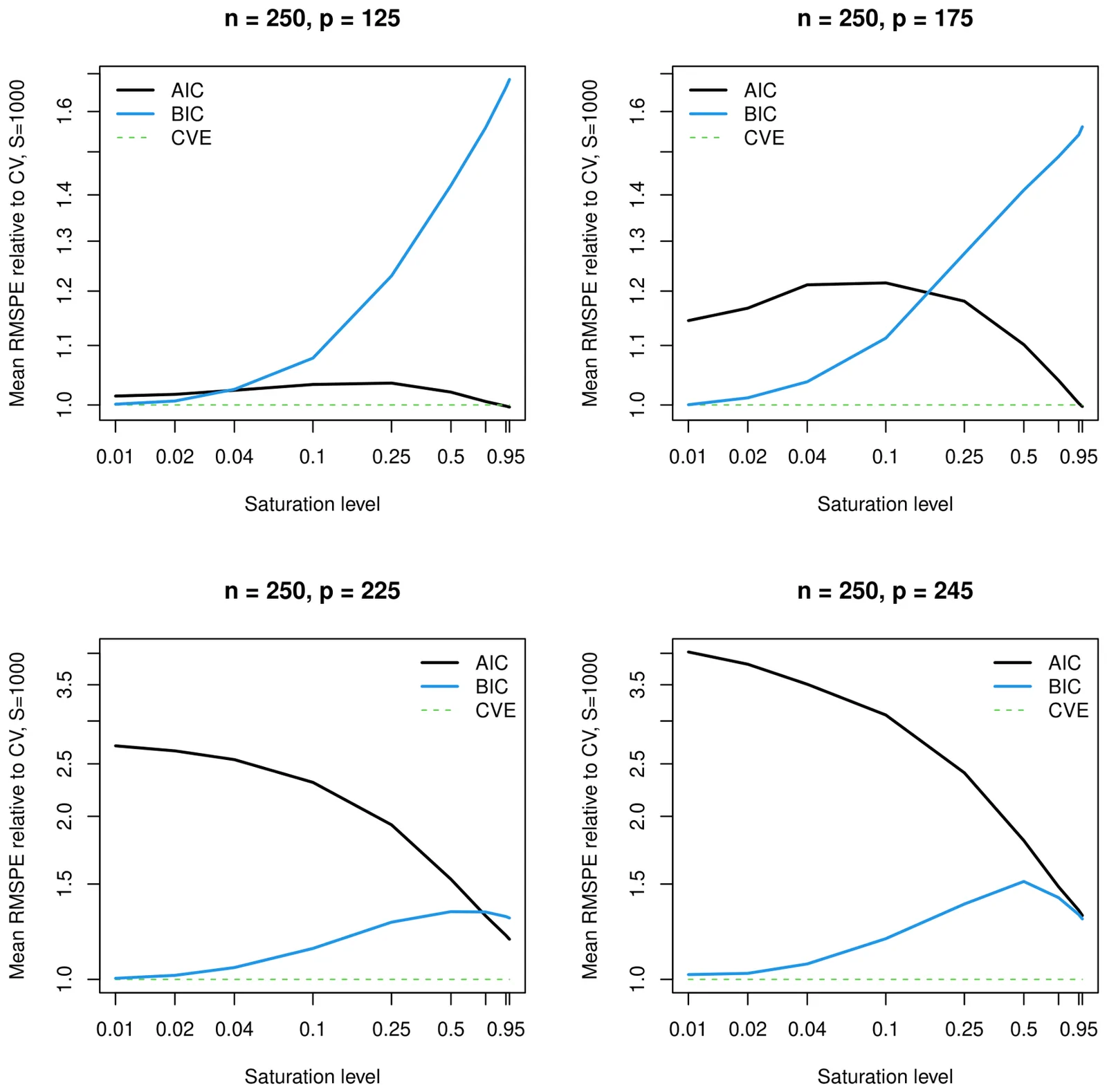
Predictive performance of lasso models tuned by AIC, BIC, or cross-validation (CV) under varying true saturation levels. Each panel fixes the sample size at n=250 and a different number of candidate predictors *p* (given in the panel title); the horizontal axis varies the true saturation level ω=s/p, the proportion of predictors with non-zero coefficients. The vertical axis reports the mean root-mean-squared prediction error (RMSPE) on held-out data relative to the CV-tuned model, averaged over S=1000 simulated datasets. BIC predicts best when the truth is sparse and AIC when it is saturated; the gap between them widens as *p* approaches *n*, while CV performs well across all saturation levels.

**Figure A2:**
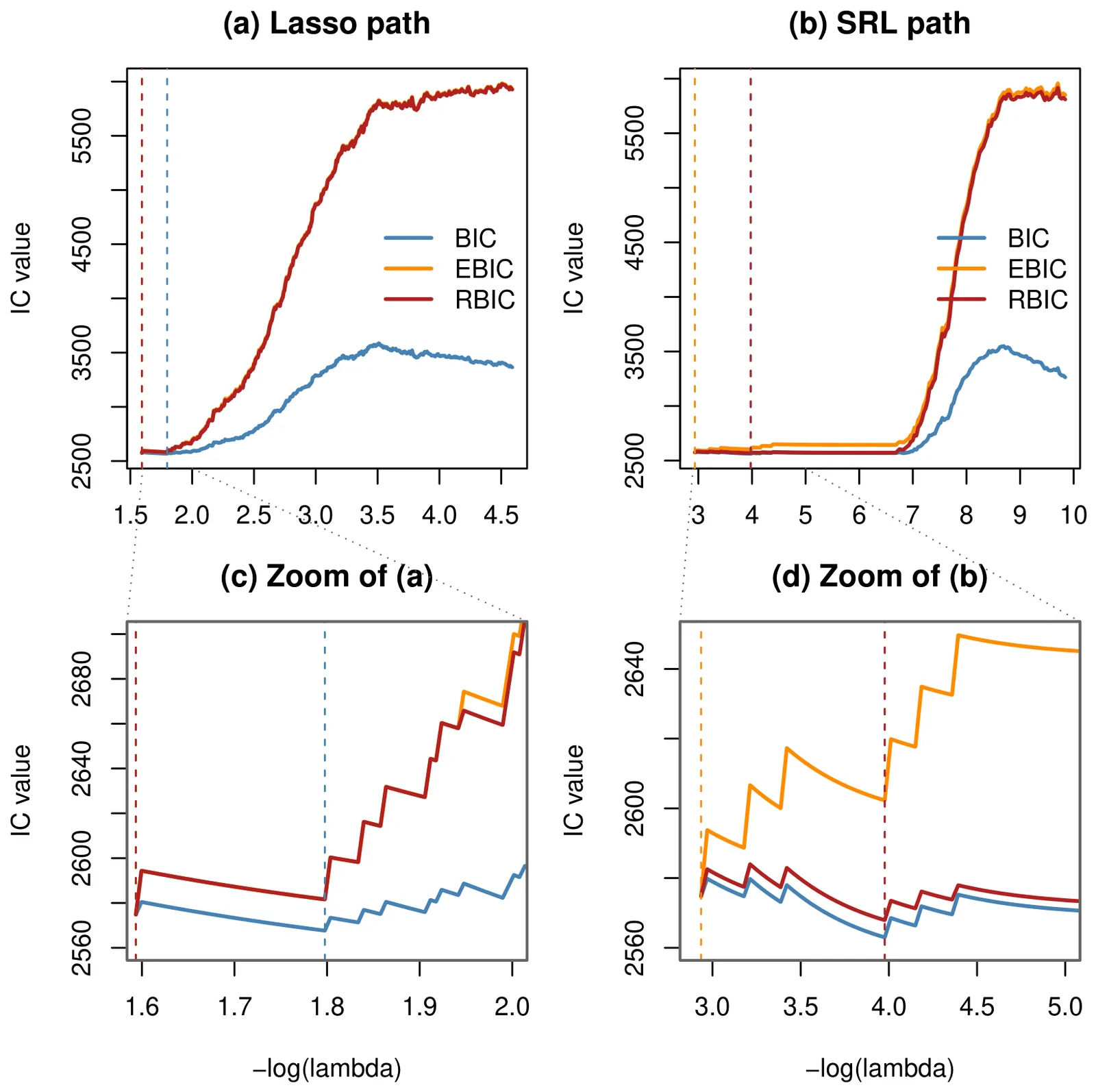
Information criteria along the SRL and standard lasso regularization paths for the Shedden lung cancer data. Panels (**a**,**b**) show the full paths; dotted lines link the lambda interval enlarged in panels (**c**,**d**) to its location on the full path. Vertical dashed lines mark the lambda that minimizes each criterion. Note that EBIC and RBIC use the same penalization for the lasso, while BIC and RBIC use the same penalization for SRL. The left-hand side of the plots represents the null model, where no covariates are active.

## Data Availability

The de-identified data for Application 1 (COVID-era ICU caregiver cohort) are distributed with the ICtoolkit R package, available at https://github.com/petersonR/ICtoolkit (accessed on 16 June 2026). The data for Application 2 are from the publicly available lung adenocarcinoma study of Shedden et al. [32]. The simulation and application code are publicly available at https://github.com/petersonR/RBIC_Manuscript_Code (accessed on 16 June 2026).

## Appendix A. Saturation-Level Simulation: Model Selection Criteria and Sparsity Levels

### Appendix A.1. Simulation Setup

In this appendix, we illustrate the finite-sample behaviors of AIC (as an exemplary efficient criterion) and BIC (as an exemplary consistent criterion) for various sparsity/saturation levels in a simulation. Specifically, for a fixed *n*, we demonstrate the following.

(1) A higher “true” saturation level requires a criterion with a lower penalty, with
  (a) BIC outperforming AIC in a setting with a low saturation level;
  (b) AIC outperforming BIC in a setting with a high saturation level.
(2) As the number of candidate variables *p* increases, the performance of AIC deteriorates.
(3) Cross-validation (CV) selects an optimal penalty based on extra-sample performance.

We set n=250, p∈ {125, 175, 225, 245}, and the number of signal variables s|p=p×(0.01,0.02,0.04,0.1,0.25,0.5,0.75,0.95,0.99) (i.e., we vary the saturation level s/p). For each combination of these, we generate *p* covariates independently from the standard normal distribution, then generate $y=X\beta +N(0,\sigma^{2}=5^{2})$, where $\beta^{T}=\left(1_{s}^{T},0_{p-s}^{T}\right)$. There are *s* signal variables and p−s null variables for each simulation. Since the number of non-zero coefficients is changing across levels of *s*, the signal-to-noise ratio ($\mathrm{SNR}=\beta^{T}Var\left(X\right)\beta /\sigma^{2}$) changes as well for different sparsity levels (Table A1). Sparse settings are typically settings where the SNR is low, whereas truly saturated settings typically indicate a higher SNR, so we believe this reflects a useful shift. However, we also investigate what happens when the SNR is fixed. We henceforth denote the saturation level as ω, where ω=s/p.

We use the lasso search method and select the tuning parameter λ according to AIC and BIC to compare their performance, relative to CV as a baseline. We utilize the lasso as a model selection tool (as opposed to a stepwise procedure) for its computational efficiency. We also generate 10∗n new observations and responses, calculating each model’s RMSPE over the N=10n held-out observations as a gold-standard for comparison. While it does not directly consider whether the model has the right covariates or not, model mis-specification (either under- or over-specification) will still inflate the RMSPE in cases where the model is badly specified. To showcase our points (1), (2), and (3) above, we plot the mean RMSPE across 1000 simulations by the saturation level in a series of plots.

**Table A1:** Design of the saturation-level simulation. Rows index the true saturation level ω=s/p and columns the number of candidate predictors *p*, with the sample size fixed at n=250. The upper block reports the number of true signal variables *s*, and the lower block the resulting effective signal-to-noise ratio (SNR); sparser settings (smaller ω) correspond to lower SNR. These configurations are used to compare AIC, BIC, and cross-validation for tuning lasso models.

	p=125	p=175	p=225	p=245
**Number of Signal Variables**				
ω=0.01	1	1	2	2
ω=0.02	2	3	4	4
ω=0.04	5	7	9	9
ω=0.1	12	17	22	24
ω=0.25	31	43	56	61
ω=0.5	62	87	112	122
ω=0.75	93	131	168	183
ω=0.95	118	166	213	232
ω=0.99	123	173	222	242
**Effective SNR**				
ω=0.01	0.04	0.04	0.08	0.08
ω=0.02	0.08	0.12	0.16	0.16
ω=0.04	0.20	0.28	0.36	0.36
ω=0.1	0.48	0.68	0.88	0.96
ω=0.25	1.24	1.72	2.24	2.44
ω=0.5	2.48	3.48	4.48	4.88
ω=0.75	3.72	5.24	6.72	7.32
ω=0.95	4.72	6.64	8.52	9.28
ω=0.99	4.92	6.92	8.88	9.68

### Appendix A.2. Results

In Figure A1, we observe that, as expected, there is a large discrepancy in the predictive quality of the model fits selected by AIC and BIC depending on the saturation level, but that this manifests differently for different *n* and *p* combinations. When p=125 (top-left), AIC and BIC perform relatively similarly for sparse settings, but as the saturation level increased, AIC outperforms BIC. As p→n, however, the performance of AIC deteriorates to the point where BIC greatly outperforms AIC in sparse settings, and there is little, if any, benefit in using AIC even in saturated settings. In all cases, (10-fold) cross-validation performs optimally at selecting the optimal λ value. Thus, we have shown in this example that the optimal penalty depends, to an extent at least, on the sparsity level (which we typically cannot know ahead of time).

A similar simulation was performed that holds the effective SNR fixed at 0.5 across all saturation levels. In this setting, very low saturation corresponds to a small number of large, easily detected coefficients, while higher saturation distributes the same total signal across many small coefficients near the noise floor. These supplemental simulations again showed AIC performing increasingly poorly as *p* approached *n*. However, once saturation exceeded roughly 0.1, BIC remained surprisingly competitive with AIC: with individual coefficients near the noise floor, AIC’s looser penalty admitted weak signals that contributed little to prediction, while BIC’s stronger penalty trimmed to the detectable subset. As a result, the criterion-to-criterion contrast was less pronounced than in the main simulation.

## Appendix B. Regularization-Path Analysis for the Lung Adenocarcinoma Data

This appendix reports the regularization-path analysis for the Shedden lung adenocarcinoma data of Application 2, complementing the forward-stepwise results in the main text.

For the regularization-path approaches, we fit penalized Cox proportional hazards models using the lasso penalty via ncvsurv. The Sparsity-Ranked Lasso (SRL) modifies the standard lasso by assigning group-specific penalty factors $\lambda_{j}=\lambda \cdot \sqrt{p_{g}}$, where *g* denotes the group to which a predictor *j* belongs. Under this scheme, the $p_{1}=7$ clinical predictors each receive a penalty factor of $\sqrt{7}\approx 2.6$, while the $p_{2}=22,283$ genetic predictors receive a factor of $\sqrt{22,283}\approx 149.3$, giving clinical variables roughly a 56-fold advantage in the penalization scale. Combining the SRL search with RBIC selection along the regularization path brings ranked sparsity into both stages of the model selection process: the search and the selection.

### Appendix B.1. Regularization Paths

Table A2 presents the (Search × Selector) grid: Lasso and SRL each crossed with BIC, EBIC, RBIC, and 10-fold CV. When using the standard lasso penalty, the information criteria-based selectors produced highly sparse models. BIC selected a one-parameter model containing a single genetic covariate, while EBIC and RBIC both produced null models. CV-based selection, which does not penalize for model complexity, produced a much larger model with 29 covariates (28 genetic; 1 clinical).

**Table A2:** Models selected along the penalized Cox regularization path for the Shedden lung adenocarcinoma data (n=442), for each combination of search strategy (standard lasso vs. Sparsity-Ranked Lasso) and selection criterion (BIC, EBIC, RBIC, or 10-fold cross-validation). λ is the penalty value chosen by each selector; Total, Clinical, and Genetic give the number of selected predictors overall and within the clinical and genetic modality groups.

Selector	λ	Total	Clinical	Genetic
**Lasso**				
BIC	0.1657	1	0	1
EBIC	0.2032	0	0	0
RBIC	0.2032	0	0	0
CV	0.1096	29	1	28
**Sparsity-Ranked Lasso**				
BIC	0.0188	3	3	0
EBIC	0.0531	0	0	0
RBIC	0.0188	3	3	0
CV	0.0009	18	7	11

SRL applies differential penalty factors according to data modality (genetic vs. clinical) and therefore produced a slightly different pattern of selection. Both BIC and RBIC yield the same small (p=3) interpretable model consisting entirely of clinical covariates. EBIC selection yielded a null model, indicating that EBIC remained overly conservative in its penalization relative to BIC and RBIC, even when paired with SRL. CV-based selection under SRL yielded a more moderate model than its lasso counterpart, with a dramatic reduction in the total number of selected covariates alongside an increase in clinical representation (all 7 clinical covariates selected compared to 1 under the standard lasso).

These results illustrate a key limitation of the standard lasso in this high-dimensional multimodal setting: when the clinical and genetic predictors are penalized equally, the sheer scale of the genetic predictor space renders individual clinical covariates nearly impossible to justify for inclusion under any information criterion. SRL addresses this directly by incorporating modality-specific penalty factors, allowing clinically relevant predictors to compete for inclusion without being crowded out by the much larger genetic predictor pool.

### Appendix B.2. IC Path Figure

Figure A2 displays BIC, EBIC, and RBIC along the regularization path for both SRL and standard lasso, starting on the left from the null model. These plots show that these methods are functioning appropriately in that they are selecting covariates to be added into models as penalization decreases, which steadily increases the IC values presented on the vertical axis.

Of note, BIC values appear to reach a peak and then steadily decrease as more parameters are introduced into the model, indicating that BIC does not sufficiently penalize model complexity in this high-dimensional setting. This is a well-documented limitation of standard BIC when the number of candidate predictors is large relative to sample size. In contrast, EBIC and RBIC maintain a more stable plateau or continue to increase across the regularization path, suggesting they are better suited for variable selection in this context. EBIC and RBIC exhibit qualitatively similar IC path behavior, and all three criteria achieve their minima in a relatively sparse region of the path before complexity penalties begin to dominate.
